# Perfectionism Profiles and Anger Responses: The Relevant Role of Self-Esteem in Athletes of Professional Quarries

**DOI:** 10.3390/ijerph17041416

**Published:** 2020-02-22

**Authors:** Antonio Jesús Muñoz-Villena, Manuel Gómez-López, Juan González-Hernández

**Affiliations:** 1 Department of Physical Education, Sport and Human Movement, Autonomous University of Madrid, 28049 Madrid, Spain; aj.munnoz@gmail.com; 2 Department of Sport Psychology Fútbol Base ElcheCF, Sociedad Anónima Deportiva (SAD), 03208 Elche, Spain; 3 Department of Physical Activity and Sport, Faculty of Sport Sciences, University of Murcia, 30720 Santiago de la Ribera, Spain; 4 Campus of International Excellence “Mare Nostrum”, University of Murcia, 30720 Santiago de la Ribera, Spain; 5 Department of Personality, Evaluation and Psychological Treatment, University of Granada, 18071 Granada, Spain; jgonzalez@ugr.es

**Keywords:** anger expression, personality, perfectionist efforts, sports technology

## Abstract

Perfectionism is a multidimensional personality trait characterized by effort and rigidity in setting high personal standards, accompanied by an excessive tendency toward critical assessments, which plays an important role in cognitive, behavioral, and emotional functioning. During adolescence, personality is built on a fundamental pillar—self-esteem—which plays an important role in sports practice when it comes to achieving the best possible performance. Anger has an emotional component that, interpreted in an unadaptive way, interferes with sports performance. The aim of this study is to assess differences according to self-esteem and perfectionism indicators and anger expression and management in young athletes. The sample included 229 male athletes to the quarries of professional sports with ages between 13 and 17 years. They were administered the Multidimensional Perfectionism Scale, the State-Trait Anger Expression Inventory for Children and Adolescents, the Rosenberg Self-esteem Scale, and a socio-demographic questionnaire. Predictive analysis showed that low personal standards and high levels of organization (indicators of adaptive perfectionism) acted as predictors of state anger, while those showing high personal standards predicted high anger management in athletes with high self-esteem. High personal standards predicted lower indicators of trait anger in athletes with low self-esteem. The results revealed the influence of high self-esteem as a protective factor in the predictive relationship among anger traits and personal standards. The study describes the relationship of these variables in the belongings of young male footballers (under high sport pressure), showing the need to take care of the athletes’ self-esteem in sport environments through prevention programs that include psychological and social resources training systems.

## 1. Introduction

Over the last few decades, a series of studies carried out with athletes [1,2,3] and with other performance-related populations [4,5,6,7] have promoted advances in the study of perfectionism by relating it to functional and adaptive aspects. Thus, perfectionism is now defined as a multidimensional personality trait characterized by effort and rigidity in setting high personal standards, accompanied by an excessive tendency toward critical assessments [6,7,8], which plays an important role in cognitive, behavioral, and emotional functioning [8,9,10]. Being a multidimensional construct, it has been studied using different models and measures, and thus dimensions based on intrapersonal and/or interpersonal aspects have been found. According to previous research [11,12,13] as to how perfectionist dimensions participate in the behavioral regulation individually, perfectionism has been associated with adaptative or maladaptive aspects. Perfectionism has been associated with adaptive aspects described by factors such as achievement expectations (the tendency to set high goals and carry out excessive self-evaluation) and organization (emphasis on the importance of order and coherence in aims proposed). On the other hand, the maladaptive aspects are described by factors such as external expectations (understood as athletes’ perception of their family and coaches’ high expectations) and the fear of making mistakes (excessive concern in relation to mistakes and failure, which causes doubts and ruminations about the quality of their performance).

**Figure 1:**
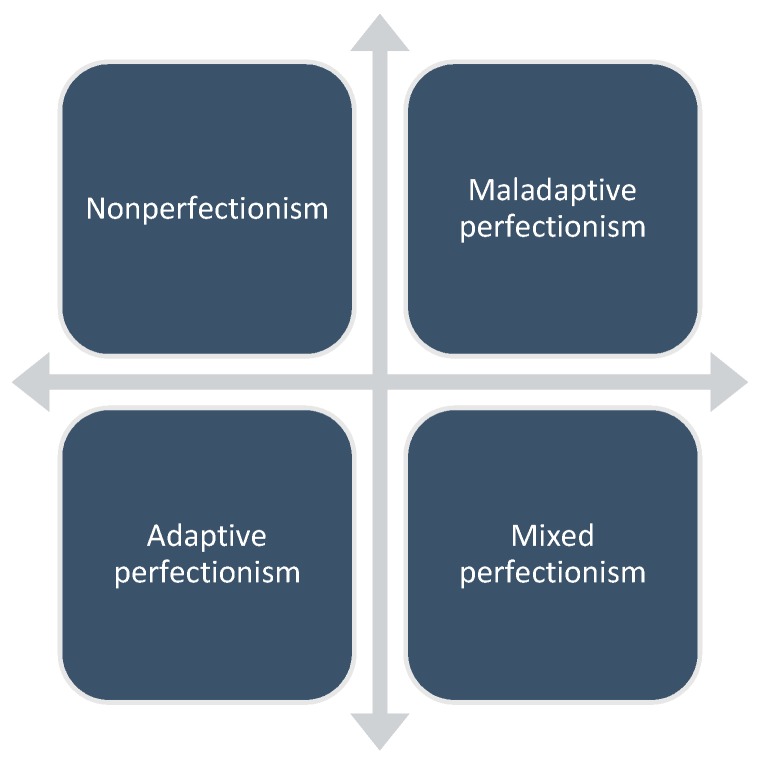
The 2 × 2 model of perfectionism (Gaudreau and Thompson, 2010).

On previous dimensions, Gaudreau and Thompson (2010) have created a 2 × 2 model of perfectionism (Figure 1), where they have differentiated four categories: nonperfectionism (low personal standards, low evaluative concerns), maladaptive perfectionism (low personal standards, high evaluative concerns), adaptive perfectionism (high personal standards, low evaluative concerns) and mixed perfectionism (high personal standards, high evaluative concerns).

In young people’s sporting performance, perfectionism is seen as a predictor of adaptation and psychological well-being when figures in the sporting context (e.g., coaches or parents) and themselves focus their efforts on how to realistically achieve the proposed goals and provide appropriate support [13,14,15,16]. In contrast, perfectionism predicts psychosocial maladjustment when the environment is contradictory (e.g., different rules of behavior at home and in the sports context) or too much rumination or low emotional self-regulation are part of the beliefs of athletes [16,17]. Thus, when the approval and affection received by a competing athlete is conditioned by the attainment of unrealistic goals or performance standards, associations with emotional difficulties and self-esteem linked to dysfunctional thoughts have been found [2,17].

Personality is fundamentally built on self-esteem, its stability shares a common development and interacts with beliefs about how to achieve goals, both autonomously and guided by other significant figures (e.g., parents, coaches, teachers, etc.) [18,19]. Self-esteem is defined as the positive or negative assessment that athletes make about themselves and their identity. It also expresses the ability to feel competent, capable, and successful that derives from a persons’ perception of dissonance or similarity in relation to the achievement of ideal personal values or standards. Thus, the level of self-esteem can be similar to perfectionism in the process of comparison between values and discrepancies. A minimum distance perceived between the “ideal self” and the “real self” results in healthy self-esteem. In this process of comparison, self-criticism manifests a radical importance, which, in individuals with low self-esteem, is usually excessive and leads to permanent dissatisfaction [20].

Therefore, as previous studies have shown [2,21], the sports environment is an ideal context for studying self-esteem in adolescence and its relationship with psychological processes when taking into account their enhancing or threatening status. Competition and the agents that surround sport have a high emotional impact on young people that sometimes leads to the abandonment of sport activity at an early age [22]. At the same time, the literature has shown that high levels of self-esteem are associated with health and psychological well-being [23], pressure or anxiety management [2], and sports performance [22]. Ichraf et al. [21] studied the association between high levels of self-esteem and low levels of anxiety in young athletes in individual sports and observed that self-esteem could function in terms of emotional control or as a protective mechanism against unadaptive situations.

Anger has an emotional component that, when interpreted in an unadaptive way, interferes in a negative way with the learning derived from sports practice. However, anger can make it possible to compete while following sport rules and social behavior with high intensity and activation, but without intent to harm or injure opponents [24]. Furthermore, different studies have suggested that adequate anger management could become a protective health factor [25,26], so that anger would have a variety of adaptive functions, including the regulation of internal psychological and physiological processes related to self-defense, along with the regulation of interpersonal and social behaviors. This emphasizes the importance of ascertaining the internal mechanisms that activate or regulate self-perception and the expression of unadaptive anger, since the latter acts as a mediator between frustration and aggression (behavior aimed at causing harm to people or things).

The objective of the study is to assess the differences between levels of self-esteem and indicators of perfectionism and anger expression and management in young athletes. The starting hypothesis are as follows:

**Hypothesis** **1.** *Athletes with higher levels of self-esteem will show higher values in adaptive perfectionism indicators (organization and personal standards) and anger management.*

**Hypothesis** **2.** *Athletes with low levels of self-esteem will show higher values in unadaptive perfectionism indicators (external expectations and fear of making mistakes) and state and trait anger.*

**Hypothesis** **3.** *Athletes with no perfectionism and adaptive perfectionism profiles will be associated with lower anger and high self-esteem.*

**Hypothesis** **4.** *Athletes with mixed perfectionism profiles will be associated with anger and lower self-esteem.*

## 2. Materials and Methods

### 2.1. Participants

The sample consisted of 229 male teenage athletes to the quarries of professional sports from different cities of Spain, selected according to accidental sampling. The age range of athletes was between 13 and 17 years old (M = 14.43; SD = 1.37). Their age distribution was 13 (34.5%), 14 (23.6%), 15 (17.5%), 16 (13.5%), and 17 (10.9%).

This study was carried out in accordance with the ethical guidelines of the American Psychological Association (APA). The protocol was approved by the Bioethics Committee of the Murcia University (ID: 1494/2017). All subjects gave written informed consent in accordance with the Declaration of Helsinki [27].

### 2.2. Measurement Instruments

*Socio-demographic questionnaire*. In terms of sample description, the previous questions cover age, the sport in question, and the level at which it was practiced together with the number of training sessions per week.

*Perfectionism*. We used the Multidimensional Perfectionism Scale adapted and validated for the Spanish context by Carrasco et al. [8] from the original Frost Multidimensional Perfectionism Scale (FMPS; [28]) to measure perfectionism. The scale includes 35 items that converge into four first-order factors (Expectations of Achievement, Organization, Fear of Mistakes, and External Influences), two second-order factors (Adaptive Perfectionism and Unadaptive Perfectionism), and one third-order factor (Global Perfectionism). The responses were distributed on a Likert scale from 1 (in total disagreement) to 5 (in complete agreement). This study analyzed the third order structure, and we obtained consistent sample reliability (Table 1); Cronbach’s Alpha was 0.87.

*Anger expression and management*. We used the State-Trait Anger Expression Inventory for Children and Adolescents (STAXI-CA) adapted and validated by Del Barrio, Aluja, and Spielberger [29] for Spanish children and adolescents from the original State-Trait Anger Expression Inventory for Adults (STAXI; [30]) in order to measure anger expression and management. This inventory includes 32 items converging in three first-order factors and two second-order factors: state anger (feeling “I’m furious” and physical and verbal expression “I feel like cursing”), anger trait (temper “I am bad-tempered” and reaction “it makes me angry to be late because of the others”), and anger expression (internal expression “I hide my feelings”, external expression “I show my anger”, and anger management “When I lose control, I can restrain myself”). The responses were distributed on a 1–3 Likert scale, whose limits are little and a lot. This study obtained sample reliability; Cronbach’s alpha was 0.79.

*Self-esteem*. The Rosenberg Self-Esteem Scale (RSS) adapted and validated by Martín-Albo, Núñez, Navarro, and Grijalvo [31] was applied to the Spanish population for the global measurement of self-esteem from the original Rosenberg Self-Esteem Scale (RSES; [32]). This scale consists of ten items (five positive and five negative). The participants scored their level of agreement with each item, using a Likert scale and four alternatives ranging from 1 (strongly disagree) to 4 (strongly agree). Cronbach’s alpha for this sample was 0.71.

### 2.3. Procedure

A protocol of action was drawn up in order to proceed in the same way in each of the sports environments. First, an interview was scheduled with the coach, technician, or manager of the Federation or Club in question in order to request the necessary authorization to administer the set of questionnaires. Once the permission was granted, a visit was agreed to, always during training sessions. Since group administration was prioritized, the protocol applied was as follows: (a) an explanation of the objectives of the study; (b) expected results; and (c) to protect anonymity, participants gave consent and were given confidentiality and anonymity in accordance with the ethical guidelines of the American Psychological Association (APA). All participants were asked for an informed consent signed by the parents/guardians, which detailed the main characteristics of the study, the relevance of the data for the improvement of existing knowledge, as well as compliance with privacy and ethics norms [27]. Subsequently, each athlete received a full and numbered questionnaire, and an evaluator was always present to solve potential doubts.

### 2.4. Data Analysis

Data coding and processing was carried out using SPSS Statistics 22.0 for Windows. Perfectionism profiles of the sample were established by segmenting scores in personal standards and evaluating concerns according to the Kolmogorov–Smirnov test and categories 2 × 2 model. We carried out internal reliability analyses of the measures used (Cronbach’s alpha), a descriptive analysis (mean and standard deviation), comparative measurements (Student´s t-test and Cohen´s d for effect size), and the Kolmogorov–Smirnov test for normality. A stepwise multiple regression for the predictive relationships by level of self-esteem (Dependent Variable: anger dimensions; Independent Variable: perfectionism) was carried out; finally, univariant analysis of variance (ANOVA) over anger expression variables and self-esteem, according to perfectionist profiles (under 2 × 2 Perfectionism Model), was carried out.

## 3. Results

Table 2 shows the descriptive statistics (mean and standard deviation) together with the normality tests of the variables studied. The Kolmogorov–Smirnov test shows that the variables follow a normal distribution, thus allowing the use of parametric tests. Statistically significant differences between athletes with low and high self-esteem were obtained for personal standards and fear of making mistakes. Furthermore, the high self-esteem category shows that the mean scores of the personal standard dimensions and anger management have higher values when compared to the low self-esteem category.

In Table 3, the results of the predictive analysis show that low personal standards and a high orientation towards organization predicts state anger when there is high self-esteem. By contrast, high personal standards predict anger management when self-esteem is high, while low personal standards predict anger trait when self-esteem is low.

In Table 4, the results of the ANOVA analysis show differences according to personality profiles in anger state (F = 3.42; *p* = 0.01) and self-esteem (F = 8.01; *p* = 0.00). The highest average score in anger state occurs in maladaptive perfectionism, while the lowest average score occurs in adaptive perfectionism. On the other hand, it is possible to highlight the same in self-esteem and average scores according to perfectionism profiles.

## 4. Discussions

The objective of this research was to assess the differences in a sample of young athletes in terms of anger expression and management according to their level of self-esteem and indicators of perfectionism. In relation to this objective, it is worth highlighting that no other similar study has been published so far, making this study innovative, given that it combines self-esteem, perfectionism, and anger expression variables.

On the basis of the results found, the first hypothesis was confirmed as athletes with high self-esteem had the highest scores in the adaptive perfectionism dimensions, in line with the results in previous research [2]. Specifically, those athletes with high levels of self-esteem had higher scores in the perfectionism sub-dimension of personal standards. These results are in line with previous research in which self-esteem is considered an important mediator between functional perfectionism and other psychological variables [33,34]. It is relevant for an athlete to continue to see him or herself as successful and to accept personal and environmental limitations when there are difficulties in achieving the objectives set. Having the ability to strive to meet a challenge along with the desire to reach it reinforces self-esteem and the possibility of engaging in innovative activities.

The results did not confirm the second hypothesis, in which lower self-esteem scores were expected to relate to dysfunctional perfectionist indicators [2,17] and anger expression. The unadaptive effects of perfectionism in the form of anxiety reactions [35] or lack of emotional control [36] raise, according to the literature, the need to promote balance and work in relation to the self-esteem of young athletes in training processes. This balance involves the socializing agents (family and coach) who are relevant in the development and maintenance of functional perfectionist tendencies; thus, it is necessary to promote and facilitate appropriate response resources in unadaptive sports situations that generate conflicts at the individual level (beliefs or disordered behaviors) and/or social levels [37,38].

Depending on the level of self-esteem, different predictors of perfectionism indicators were found in relation to anger expression and management [21]. In those athletes with high self-esteem, indicators of functional perfectionism turned out to be good predictors of anger control and state anger. Furthermore, personal standards negatively predicted state anger and positively predicted anger management, with the percentage of variance explained by each model being 28% and 22%, respectively. By contrast, athletes with low self-esteem showed that their personal standards negatively predicted trait anger, i.e., recurrent episodes of anger due to perceiving a series of situations as provocative (e.g., competitive situations).

Finally, the results have shown differences in anger and self-esteem scores according to the perfectionist profiles in the model of Gaudreau and Thompson (2010). The results confirmed the third hypothesis, identifying a positive significant relationship between personal standards and self-esteem [17] and anger [39]. By contrast, the results confirmed the fourth hypothesis, confirming previous research and identifying a positive significant relationship between evaluative concerns and anger [39,40]. As a result, mixed or maladaptive perfectionist athletes may have a greater predisposition to experience feelings such as anger as a consequence of their frustrated expectations and their tendency to judge themselves harshly [41,42]. On the other hand, the results have showed that adaptive and non-perfectionist athletes have higher self-esteem scores than other perfectionism profiles. Concern for mistakes is positively associated with the tendency to make internal attributions, while athletes with high personal standards are associated with a greater probability of attributing performance to internal causes and stable elements [17,41].

## 5. Conclusions

In conclusion, the results show that regardless of low or high self-esteem, if athletes and those around them are able to regulate personal standards, planning, and organization factors, it should be possible to control anger expression [43,44,45]. Therefore, the functional capacities of an athlete through personal standards and order are better predictors of anger management than external influences exerted by parents and coaches. These findings have made it possible to discover those regulatory mechanisms for an athlete’s performance that can promote psychological coping strategies in the face of unknown situations. The study of these types of variables in the sports field will allow sports psychologists to design prevention and/or stimulation of adaptive behavior programs.

In future studies, given the importance of these concepts for young people’s development, it will be necessary to conduct research with a sample of female athletes to analyze the moderating effect of different levels of self-esteem on perfectionism and anger management. This is one of the limitations of this study; there were numerous difficulties when it came to getting access to female athletes’ samples given that this population had recently participated in another research project. Furthermore, in view of the importance of self-esteem levels on perfectionism indicators and consequently on anger expression and management, it would be useful to have broader, homogeneous samples covering different ages through longitudinal studies to analyze whether there are variations through adolescence. In the same way, the variability of the samples should be generalized to samples of high-performance athletes and any other level of sport, not only those in the early stages of training. To this aim, more sports-specific instruments should be used as well as more complex statistical techniques, such as mediation.

Regardless of these proposals for further research, it is clear that perfectionism includes functional and adaptive aspects based on individual characteristics such as self-esteem, which have an impact on anger management. Thus, a line of research has been opened that can contribute to explaining athletes’ behaviors and emotional management in relation to their sports goals, as well as the influence of other hidden psychological variables (individual and contextual) that may be exerting an important or rather significant effect on athletes’ responses, and therefore on their beliefs, their performance, their motivations, and their learning.

## Figures and Tables

**Table 1:** Descriptive statistics of the sample.

	α
**Perfectionism**	0.87
Organization	0.87
Personal standards	0.74
Fear of making mistakes	0.76
External expectations	0.78
**Anger Expression**	0.79
Trait anger	0.72
State anger	0.87
Anger management	0.87
**Self-Esteem**	0.71

**Table 2:** Descriptive statistics of the sample.

	K–S	High Self-Esteem		Low Self-Esteem		*p*	d
		M	SD	M	SD		
Organization	0.20	27.49	6.13	27.71	6.60	0.72	−0.03
Personal standards	0.20	21.74	5.42	19.75	5.50	0.00 **	0.36
Fear of making mistakes	0.20	24.10	6.55	28.07	6.89	0.00 **	−0.59
External expectations	0.20	20.93	5.60	21.54	5.80	0.30	−0.63
Trait anger	0.20	12.98	3.03	13.67	2.99	0.12	−0.22
State anger	0.20	9.33	1.92	9.49	2.04	0.70	−0.08
Anger management	0.20	15.42	3.75	14.95	3.73	0.22	0.12

Note: K–S = Kolmogorov–Smirnov; M = Mean; SD = Standard deviation, ** *p* > 0.01.

**Table 3:** Linear regression analysis (stepwise).

	Dependent Variable	Predictive Variable	R	β	t	*p*
Low self-esteem	Trait anger	Step 1: Personal standards	0.20	−0.20	−0.01	0.04 *
High self-esteem	State anger	Step 1: Personal standards	0.21	−0.21	−2.47	0.01 *
		Step 2: Personal standards organization	0.28	−0.24 0.18	−2.82 2.18	0.00 ** 0.03 *
	Anger management	Step 1: Personal standards	0.22	0.22	2.65	0.00 **

Note: * *p* > 0.05; ** *p* > 0.00.

**Table 4:** Analysis of variance (ANOVA) according to perfectionist profiles (Gaudreau and Thompson, 2010).

		Non-Perfectionism	Mixed Perfectionism	Adaptive Perfectionism	Maladaptive Perfectionism	F	*p*	ŋ
Anger management		15.07 (4.31)	15.29 (3.31)	15.57 (3.87)	15.12 (3.15)	0.17	0.91	0.00
Anger	State	9.13 (1.72)	9.75 (2.20)	8.79 (1.46)	9.87 (2.20)	3.42	0.01 *	0.04
	Trait	12.90 (3.20)	13.40 (2.84)	13.47 (2.84)	13.44 (3.05)	0.50	0.67	0.00
Self-esteem		32.31 (3.74)	31.30 (4.41)	34.11 (3.73)	30.06 (4.06)	8.01	0.00 **	0.09

Note. * *p* < 0.05; ** *p* < 0.01.
